# Monkeypox and Global Health Inequities: A Tale as Old as Time…

**DOI:** 10.3390/ijerph192013380

**Published:** 2022-10-17

**Authors:** Monica Malta, Placide Mbala-Kingebeni, Anne W. Rimoin, Steffanie A. Strathdee

**Affiliations:** 1 Institute for Mental Health Policy Research, Centre for Addiction and Mental Health, Toronto, ON M5S 2S1, Canada; 2 Faculty of Medicine, Department of Psychiatry, University of Toronto, Toronto, ON M5T 1R8, Canada; 3 National Institute of Biomedical Research, Cliniques Universitaires De Kinshasa, Kinshasa, Congo; 4 Department of Epidemiology, Jonathan and Karin Fielding, School of Public Health, University of California, Los Angeles, CA 90095, USA; 5 Department of Medicine, University of California San Diego, La Jolla, CA 92093, USA

Infectious disease outbreaks can quickly become global in what has increasingly become a closely interconnected world, influenced by what is considered to be an unprecedented era of technological, demographic, and climatic change [1]. Infectious diseases know no borders or continents, as we have seen repeatedly with HIV/AIDS, tuberculosis, influenza, COVID-19, and now monkeypox. At the outset of each new epidemic, experts remind us that poverty, inequality, and underlying social determinants of health have created the ideal conditions for the transmission of infectious diseases [2].

Existing health disparities or inequalities, worsened by the COVID-19 pandemic [3], are deepening the disproportionate burden of morbidity and mortality which is being borne by lower- and middle-income countries (LMICs) [4]. A recent study observed the highest increases in all-cause mortality from 2015 to 2019 per 100,000 inhabitants in some Latin American and Eastern European countries: Peru (590), Bulgaria (460), North Macedonia (420), Serbia (400), Mexico (360), Ecuador (350), Lithuania (350), and Russia (340) [5].

In September 2021, the World Health Organization (WHO) set an ambitious global target, calling for 70% of the global population to be vaccinated against COVID-19 by mid-2022 [6]. As of August 2022, about 80% of people in high-income countries had received at least one vaccine dose compared with only 20% of those in LMICs [7]. According to the WHO, the best treatment for mild and moderate COVID-19 patients at the highest risk of hospital admission is nirmatrelvir and ritonavir, sold under the name Paxlovid. However, 95% of Pfizer’s Paxlovid supply for the first half of 2022 had been bought up by rich countries [8].

Although the COVID-19 pandemic is far from over, a rapidly emerging epidemic of a neglected virus, monkeypox (MPXV), has alarmed the world. The first human MPXV infection was reported in 1970, in the Democratic Republic of the Congo, where it later became endemic. A zoonotic infection, MPXV has been primarily associated with rodents, which serve as reservoirs; for decades, it has been reported in tropical rainforest areas of Central and West Africa [9]. Dr. Dimie Ogoina, Professor of Medicine and Infectious Diseases at Niger Delta University in Nigeria, was the first to describe MPXV outbreaks among men who have sex with men from Nigeria [10]. Since then, MPXV spread to other African nations, becoming more prevalent in Central and West Africa [11]. Over the past few years, Dr. Ogoina tried to highlight the importance of this outbreak in Nigeria, and the possibility of outbreaks in other previously disease-free countries, with no success, until it started to affect higher-income countries.

In 2003, the first monkeypox outbreak outside of Africa was reported in the United States, and was linked to infected pet prairie dogs [12]. This outbreak led to over 30 MPXV cases in the United States [12]. For almost 20 years, MPXV was forgotten again in higher-income countries who no longer considered it a threat. However, beginning in May 2022, there was an atypical emergence of a new Clade II variant of MPXV in non-endemic, previously disease-free countries. As of 4 October 2022, almost 69,000 cases of Clade II have been reported in over 100 countries, including 68,000 in 100 countries that have not historically reported MPXV cases [13]. Thus far, the majority of these cases have occurred among adult men who have sex with men (MSM) in the United States, Western Europe, and the United Kingdom. To date, MPXV-related deaths have been rare, but have been reported in Belgium, Brazil, Cameroon, Central African Republic, Cuba, Czechia, Ecuador, Ghana, India, Nigeria, Spain, Sudan, the United States, and Mexico [13]. However, it is important to note that the true global burden of monkeypox is not known. The vast majority of LMICs suffer with a chronic shortage of resources, with limited testing and surveillance capacity. In LMICs, an unknown number of MPXV is occurring [14].

The fact that MPXV transmission is occurring rapidly outside the African region, where it has been endemic for the past 50 years, highlights a number of features that make this disease globally important. First, the end of routine smallpox vaccination in 1980, which conferred cross-protection against MPXV, is believed to have played a role in outbreaks in the DRC [15], as well as the current pandemic. As a result, it is estimated that more than 70% of the world’s population is no longer protected against smallpox, which conferred some degree of protection against other poxviruses, such as monkeypox [16]. Second, climate change [17] and food insecurity [18] have caused greater interactions between humans and animals, which increase the risk of zoonotic infections. Third, economic decline has forced many rural residents (especially from the DRC) to rely on hunting for sustenance, thus increasing exposure to animal reservoirs of MPXV [19]. Fourth, the rising global population mobility, allied with increased contact patterns in highly populated areas are of key concern [20].

In mid-2022, MPXV was declared a Public Health Emergency of International Concern (PHEIC) by the WHO [21]. However, the fact that this disease has been neglected in endemic regions for five decades highlights our failure to address the health needs of people living in LMICs. The real concern that MPXV brings is not when a PHIEC is declared, but why the world did not act preventively, improving LMIC abilities to monitor and act prior to the point of needing to declare it a PHIEC. Worldwide, but especially in LMICs, healthcare resources are even more impoverished than before the COVID-19 pandemic. The risk of a new pandemic, or new local outbreaks of MPXV, could be devastating for countries with already fragile healthcare systems.

The disparity between wealthy nations and the rest of the globe can be highlighted, once again, by the access to MPXV vaccines—currently available only in high-income countries such as Canada, the United States, and the United Kingdom. With only 16.4 million doses of the Jynneos vaccine available worldwide, and the company’s European manufacturing plant shuttered until late 2022, it remains unclear how the world could meet the rising demand [22]. We are, once again, failing the populations most in need. In this case, we are failing every single individual infected with MPXV living in LMICs, especially those who have been battling MPXV in Africa for the last five decades [23].

If worldwide responses to infectious diseases continues to be led by “if and when” those diseases reach high-income nations, we will continue witness a heavy toll of health disparities and avoidable morbidity and mortality in LMICs. According to Dr. Madhukar Pai, “global health is failing in equity, reciprocity, and genuine bi-directional partnerships” [24]. We can, and must, do better.

It is imperative to improve LMICs’ capacity to properly and quickly respond to health emergencies such as MPXV. According to a consensus statement published in JAMA, pandemic preparedness is vital [25]. This should include a systematic evaluation of the country’s experiences during the COVID-19 pandemic, trying to answer the following questions: “what worked well; what strengths can be leveraged; and what did not work well, why, and what is needed to augment infrastructure and to manifest organizational values” [25]. The Pan American Health Organization states that “a progressive transition from pandemic response to recovery will require a renewed focus on building sustainable, resilient health systems, informed by and building on the COVID-19 response” [26].

Experiences and knowledge accumulated during the COVID-19 pandemic should be leveraged. This unparalleled health crisis ignited unprecedented health innovation, as well as highly productive collaborations and coalitions [27]. However, the COVID-19 pandemic also showed that corporate greed and patent protections prevented equitable access to COVID-19 vaccines in LMICs. For example, Dr. Peter Hotez and Dr. Maria Elena Bottazzi developed a low-cost, patent-free, and highly effective COVID-19 vaccine called CORBEVAX [28]. Moreover, the production process of this protein-based vaccine is familiar to many drug manufacturers in LMICs, enabling a rapid scale-up. The vaccine was authorized in India, where around 100 million doses are being produced every month—hopefully, the first of many LMICs to start its production. However, for many LMICs, patent waivers are not the key problem—many countries still need to build vaccine production capacity. The successes, failures, and knowledge gained during the COVID-19 pandemic should inform new strategies towards responding in a more equal and sustainable way to the current MPXV pandemic.

Monkeypox started in Africa, and is rapidly spreading to several LMICs. How many persons living in LMIC have received the monkeypox vaccine? Zero. It is past time for global health to really become global.
